# Systematic identification of long non-coding RNAs with cancer-testis expression patterns in 14 cancer types

**DOI:** 10.18632/oncotarget.21930

**Published:** 2017-10-19

**Authors:** Na Qin, Cheng Wang, Qun Lu, Zijian Ma, Juncheng Dai, Hongxia Ma, Guangfu Jin, Hongbing Shen, Zhibin Hu

**Affiliations:** ^1^ State Key Laboratory of Reproductive Medicine, Nanjing Medical University, Nanjing 211166, China; ^2^ Department of Epidemiology and Biostatistics, School of Public Health, Nanjing Medical University, Nanjing 211166, China; ^3^ Jiangsu Key Lab of Cancer Biomarkers, Prevention and Treatment, Collaborative Innovation Center for Cancer Medicine, Nanjing Medical University, Nanjing 211166, China; ^4^ Department of Bioinformatics, School of Basic Medical Sciences, Nanjing Medical University, Nanjing 211116, China

**Keywords:** cancer-testis gene, long non-coding RNA, genomic instability, lung adenocarcinoma

## Abstract

Cancer-testis (CT) genes are a group of genes that are potential targets of immunotherapy and candidate epi-drivers participating in the development of cancers. Previous studies mainly focused on protein-coding genes, neglecting long non-coding RNAs with the same expression patterns. In this study, we performed a systematic investigation of cancer-testis long non-coding RNAs (CT-lncRNAs) with multiple independent open-access databases.We identified 1,325 extremely highly expressed CT-lncRNAs (EECT-lncRNAs) in 14 cancer types. Functional annotation revealed that CT-lncRNAs reactivated in cancers could promote genome instability and the malignant potential of cancers. We observed a mutually exclusive pattern of EECT-lncRNA activation and mutation in known oncogenes, suggesting their potential role as drivers of cancer that complement known mut-driver genes. Additionally, we provided evidence that testis-specific regulatory elements and promoter hypo-methylation may be EECT-lncRNA activation mechanisms, and EECT-lncRNAs may regulate CT gene reactivation. Taken together, our study puts forth a new hypothesis in the research field of CT genes, whereby CT-lncRNAs/EECT-lncRNAs play important roles in the progression and maintenance of tumorigenesis, expanding candidate CT epi-driver genes from coding genes to non-coding RNAs.

## INTRODUCTION

Cancer-testis (CT) genes are genes with an expression pattern restricted to cancer and testical tissue. Originally they were named cancer-testis antigens since CT genes encode proteins that can evoke immune responses [1, 2]. Because of their immunogenicity and aberrant expression pattern, cancer-testis antigens are commonly considered as candidate immunotherapy targets for cancer therapy [1-3].

Recently, increasing evidence has suggested that CT genes can also participate in cancer development. Maxfield et al. integrated a multi-faceted platform and proved the engagement of CT genes in tumor biology [4]. Some classic CT genes, such as MAGEA3/6 and HORMAD1, have also been shown to initiate cancers [5-7]. To make a comprehensive description of these candidate driver genes, our previous work provided a global view of CT genes in tumorigenesis [8]. Interestingly, we observed that non-coding RNAs with a CT expression pattern could also contribute to the initiation of cancer.

Long non-coding RNAs (lncRNAs) are a class of RNAs identified as transcripts with a length greater than 200 nt with little or no protein-coding potential [9]. Compared to protein-coding genes, lncRNAs are transcribed at low levels. However, their expression patterns are highly tissue-specific, and they are preferentially expressed in the testis [10], which is indicative of their potential roles in gametogenesis. Recently, lncRNAs have been shown to engage in the initiation and progression of cancers by means of diverse mechanisms, including transcriptional control and post-transcriptional processing of mRNAs [4, 11]. Although attracting more attention, most lncRNAs have not been well characterized functionally.

Thus, in this study we systematically defined cancer-testis lncRNAs (CT-lncRNAs) in 14 cancers and studied their evolutionary features. Furthermore, we defined extremely highly expressed CT-lncRNAs (EECT-lncRNAs) and investigated their association with clinical characteristics and inferred their potential functions in carcinogenesis. In addition, we also explored their potential activation mechanisms and the interactions with nearby CT genes.

## RESULTS

### Identification and general description of CT-lncRNAs

Among our previously defined high-confidence testis-specific genes, 2,598 ncRNAs showed a consistent testis-specific expression pattern in multiple databases and were classified into the C2 group. Here, we distinguished CT-lncRNAs from this group. In total, 1,354 (69.22%) of 1,956 C2 TS-lncRNAs exhibited expression (reads per kilobase per million mapped reads (RPKM)>0.1) in at least 1% of the samples of any cancer type and were considered CT-lncRNAs (Supplementary Table 1). We additionally defined CT-lncRNAs with an extremely high expression pattern to indicate potentially functional lncRNAs in cancers. As a result, 1,325 of the above defined CT-lncRNAs exhibited an extremely high expression pattern in at least one cancer type (Supplementary Table 2). The activated number of EECT-lncRNAs (median: 3-9) was far more than that of extremely highly expressed CT genes (EECTGs) (median: 0-2) in each cancer sample, and they were included in the following analysis. We then examined the expression of 609 lung adenocarcinoma specific EECT-lncRNAs in 24 lung adenocarcinoma samples and validated 51 EECT-lncRNAs (Supplementary Figure 1).

**Figure 1:**
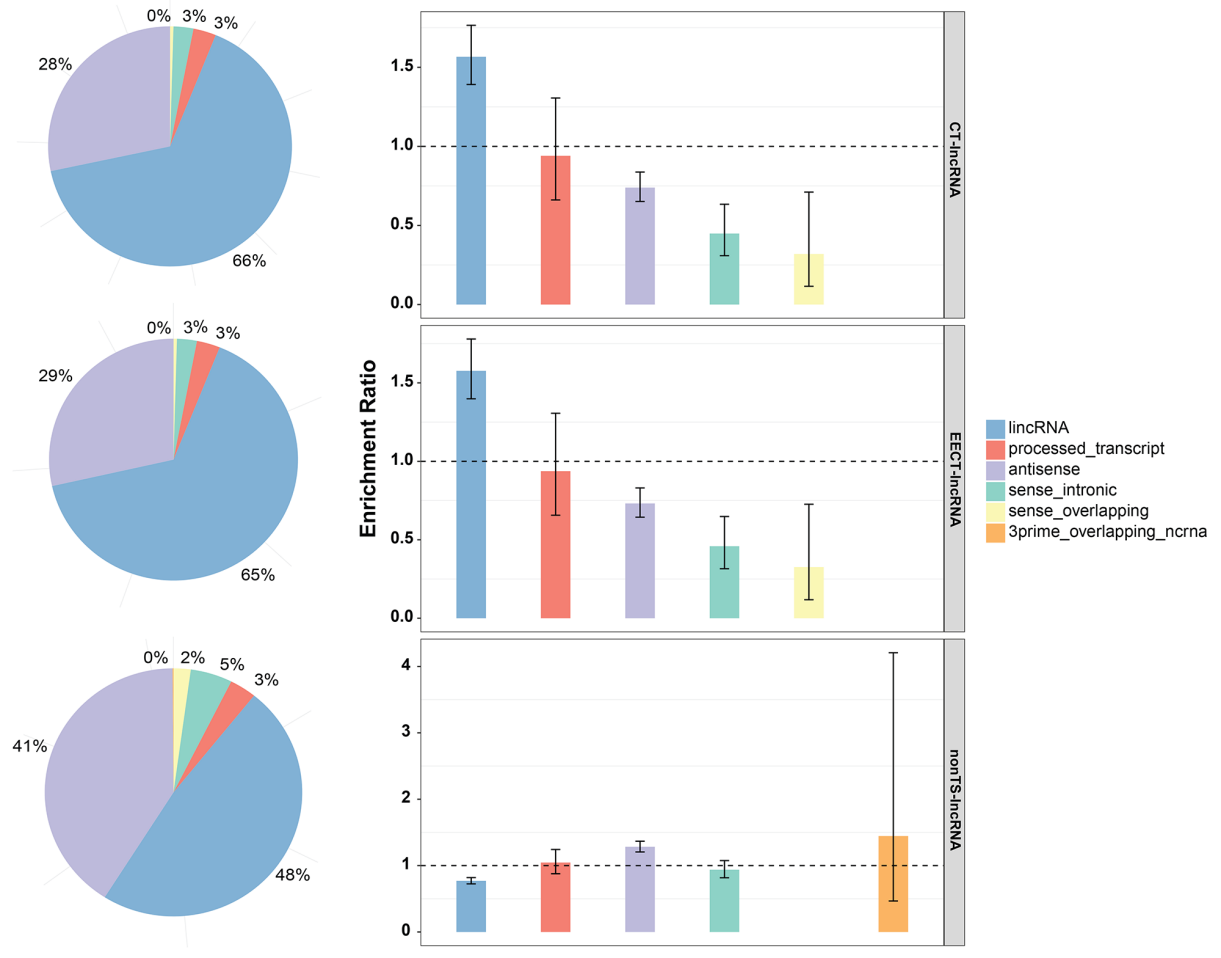
Classification of CT-lncRNAs, EECT-lncRNAs and TS-lncRNAs The bar plot represents the proportion of lncRNAs per sub-category. The error bar represents the ER confidence interval.

Among our newly defined EECT-lncRNAs, long intergenic non-coding RNA was the largest category and significantly exceeded the expected number (Figure 1). In addition, EECT-lncRNAs were not uniformly distributed across the chromosomes, and a significant deficiency in chromosome 17-19 (Supplementary Figure 2A) was observed compared to the background distribution. We further measured the conservation of the exons and promoter regions of EECT-lncRNAs with both PhastCons and PhyloP scores. Analysis result showed that protein-coding genes and lncRNAs with a CT expression pattern exhibited weaker evolutionary conservation relative to those without (Supplementary Figure 2B, 2C).

### Functional prediction of CT-lncRNAs based on co-expressed protein-coding genes

**Figure 2:**
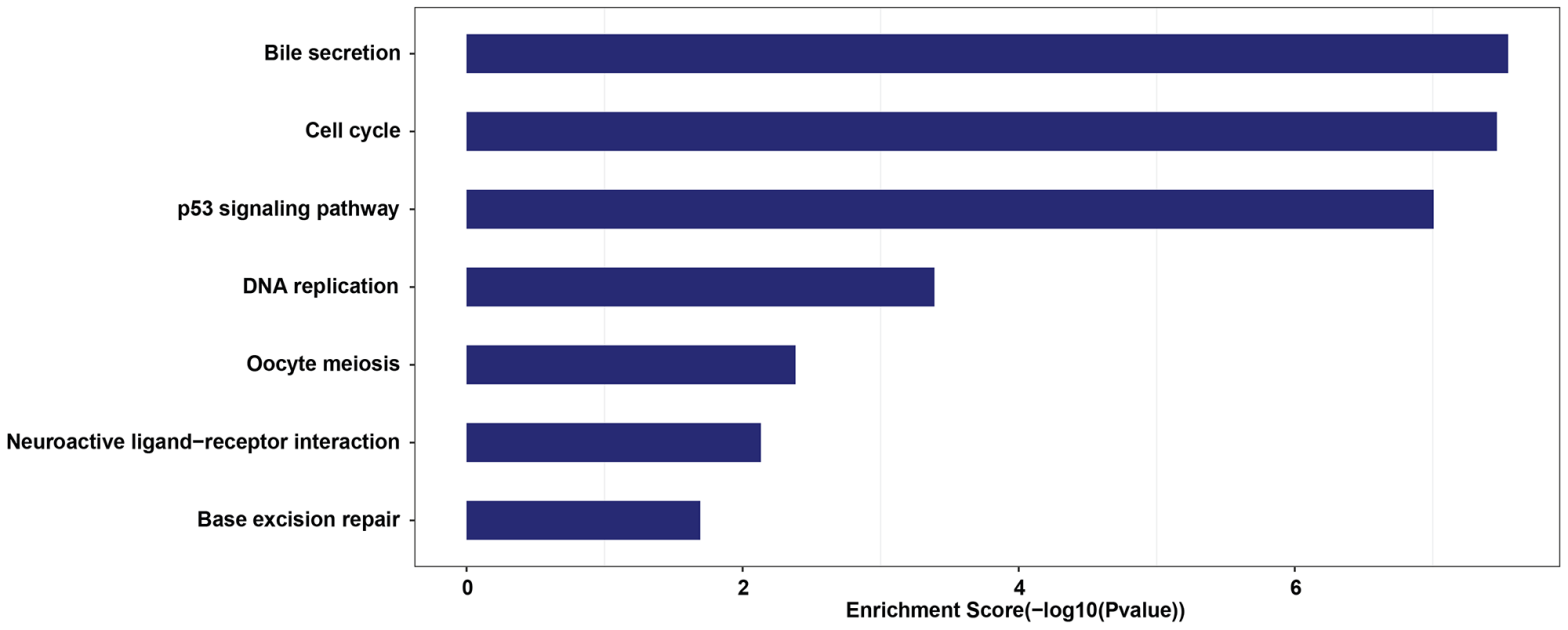
Statistically significantly enriched pathways of lung adenocarcinoma specific CT-lncRNAs

To evaluate the function of CT-lncRNAs defined in our study, we realigned and assembled the raw data of the TCGA lung adenocarcinoma samples and conducted a co-expression analysis. A total of 12,983 protein-coding genes and 8,248 lncRNAs were included in the analysis, and 12,772 protein-coding genes and 7,983 lncRNAs were successfully annotated. The pathway enrichment analysis indicated that CT-lncRNAs (Supplementary Table 3) were prone to be involved in important cancer-related pathways (p53 signaling pathway) and cell cycle-related pathways (cell cycle, DNA replication, oocyte meiosis and base excision repair) (FDR q value< 0.05) (Figure 2, Supplementary Table 4). Since such pathways can lead to aneuploidy and genome instability, we further explored the correlation between the number of activated CT-lncRNAs/EECT-lncRNAs and allele-imbalanced copy number aberrations (AICNA) (a sign of genome instability) in the sample set. Indeed, a significant positive linear correlation between AICNA and the number of activated CT-lncRNAs/EECT-lncRNAs was observed (Supplementary Figure 3), suggesting that the activation of CT-lncRNAs/EECT-lncRNAs could promote malignancy.

In an attempt to examine the relationship between EECT-lncRNAs and patient characteristics, we performed subgroup analyses by age, gender, and the American Joint Committee on Cancer (AJCC) tumor stage. Interestingly, we found that the number of activated EECT-lncRNAs increased linearly with the AJCC tumor stage, after adjustment for age, gender, and cancer type (Beta=1.06, *P*=5.43×10^−8^) (Supplementary Figure 4).

### Correlation between the EE patterns of CT-lncRNAs and mutations

**Figure 3:**
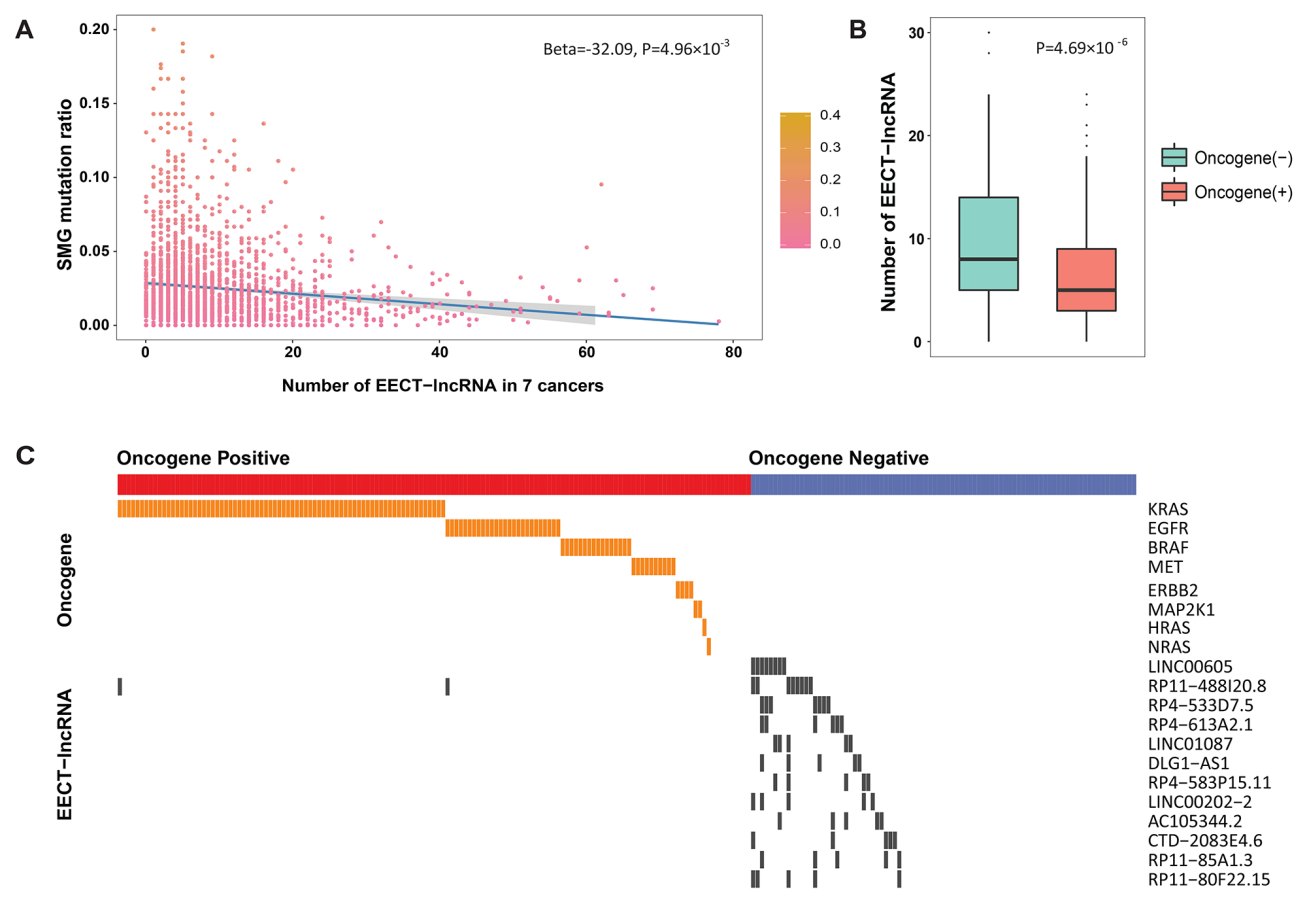
The association between SMG mutation ratio and the number of activated EECT-lncRNAs **(A)** Negative correlation between the mutation ratio of SMGs and the number of activated EECT-lncRNAs. **(B)** The total of number of activated EECT-lncRNAs is significantly higher in lung adenocarcinoma samples without clear activating oncogene mutational alterations. **(C)** Mutually exclusive pattern of EECT-lncRNA activation and oncogene mutations in lung adenocarcinoma samples.

As significant mutated genes (SMGs) are commonly considered to be the major drivers of tumorigenesis, we further explored the relationship between the expression patterns of EECT-lncRNAs and the mutation ratio of SMGs in cancer samples. Seven cancer types with more than 100 platform-overlapped samples were included. Interestingly, we observed a significant reverse association between SMG mutation ratio and the number of activated EECT-lncRNAs after adjusting for cancer type (Beta=-32.09, *P*=4.96×10^−3^) (Figure 3A).

Additionally, in 230 TCGA lung adenocarcinoma samples with clearly defined known activating mutations, patients without known mutations exhibited significantly more activated EECT-lncRNAs (*P*=4.69×10^−6^, Figure 3B). Thus, we further evaluated the correlation between the expression pattern of each EECT-lncRNA and mutations in previously defined driver oncogenes (Supplementary Table 5). This analysis revealed that 12 EECT-lncRNAs were mutually exclusive with mutations in known driver genes (Figure 3C). The above result suggests that the activation of EECT-lncRNAs may be a novel driver that complements known mut-driver genes.

### Testis-specific enhancers may be a regulatory mechanism for TS-lncRNAs/CT-lncRNAs

**Figure 4:**
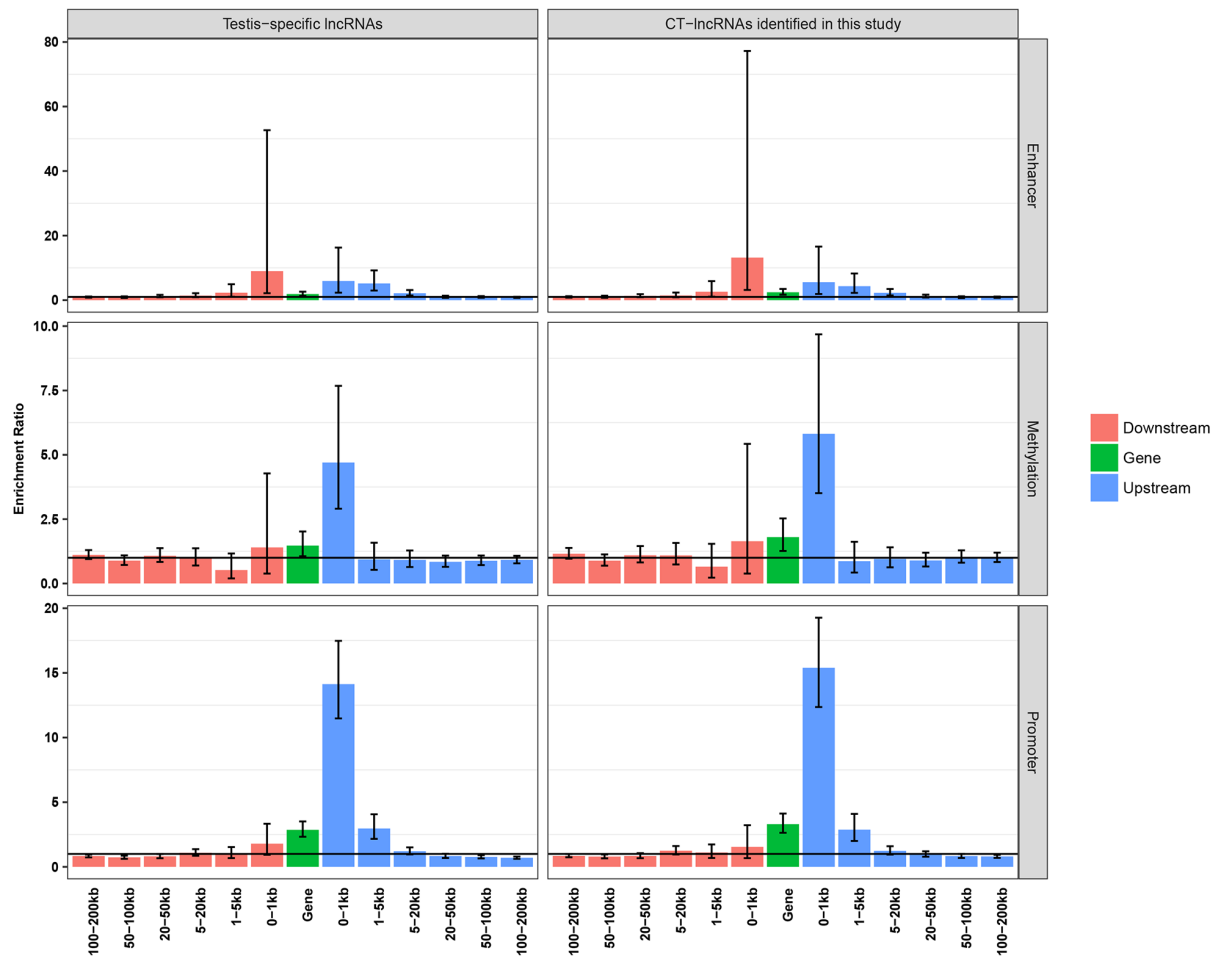
Enrichment analysis of testis-specific regulatory elements

As our previous work indicated that testis-specific regulatory elements (promoters, non-coding RNAs and methylation sites) might be potential regulators of testis-specific genes and CT genes, we further explored the correlation between testis-specific regulatory elements and the expression patterns of TS-lncRNAs/CT-lncRNAs. We found that testis-specific promoters were frequently located upstream (-100 bp to 5 kb) of TS-lncRNAs/CT-lncRNAs, while testis-specific methylation sites were more likely to be located more proximally upstream (-100 bp to 1 kb) (Figure 4). Unlike testis-specific genes/CT genes, we observed a significant enrichment of testis-specific enhancers 20 kb upstream and 5 kb downstream of TS-lncRNAs/CT-lncRNAs (Figure 4), indicating that adjacent enhancers might be another regulatory mechanism for the activation of TS-lncRNAs/CT-lncRNAs.

We then divided the TS-lncRNAs into two categories according to the presence of enhancers 20 kb upstream or 5 kb downstream to evaluate the effect of enhancers on the expression correlation between TS-lncRNAs and testis-specific genes. We assessed the correlation coefficient between TS-lncRNAs and their proximate testis-specific genes in the testis. We observed a significantly stronger correlation between these TS-lncRNAs without enhancers nearby and their proximate testis-specific genes than for those with enhancers nearby (*P*=2.57×10^−2^) (Supplementary Figure 5). This result supports a conclusion that enhancer elements may be a specific regulatory mechanism for the activation of TS-lncRNAs but not for testis-specific genes.

### EECT-lncRNAs may be activated by promoter hypo-methylation

**Figure 5:**
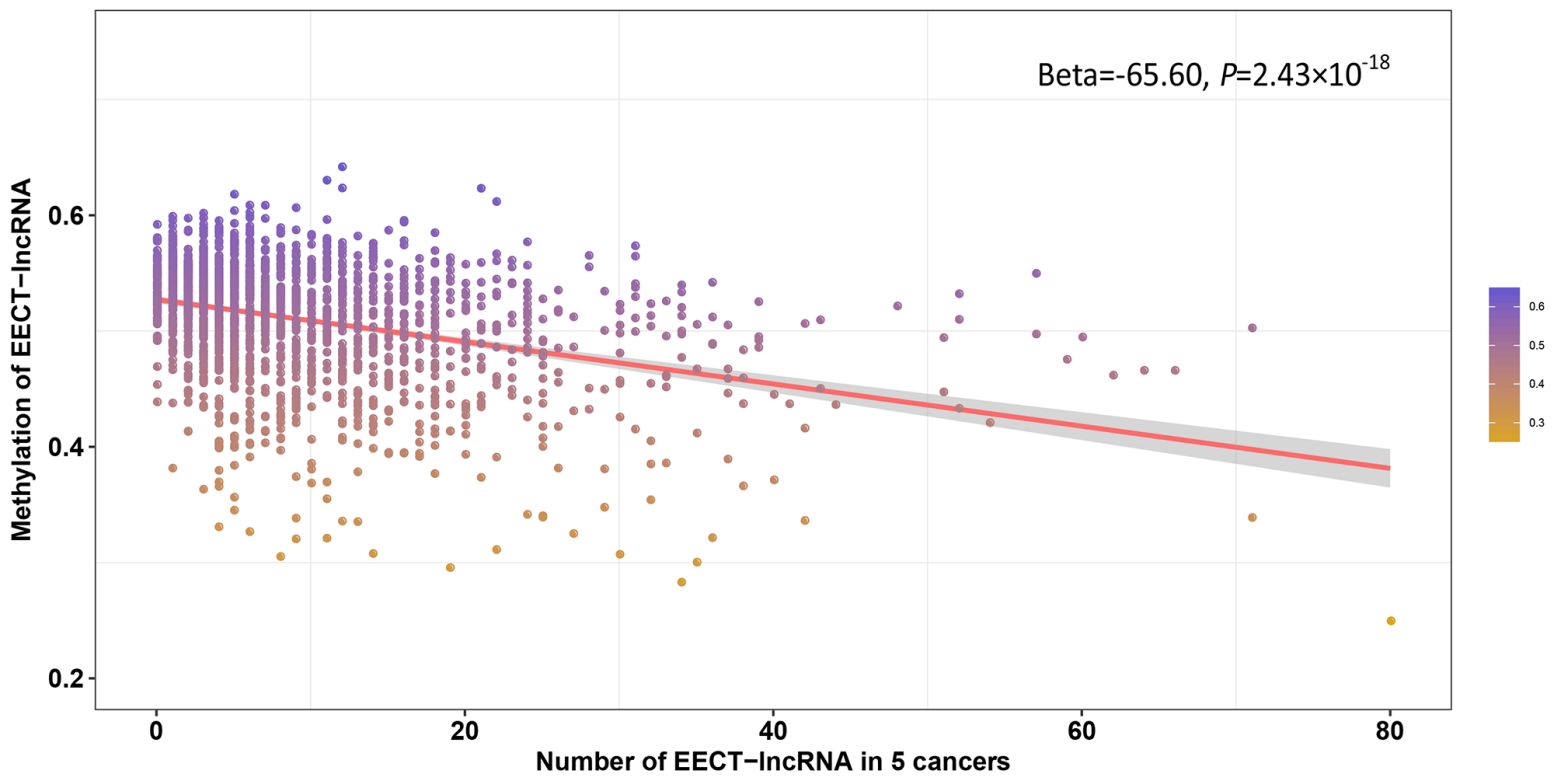
Negative correlation between the average promoter methylation level of EECT-lncRNAs and the number of activated EECT-lncRNAs

In addition to testis-specific enhancers, we attempted to explore other regulatory mechanisms of EECT-lncRNA activation in cancer. We integrated DNA methylation and expression data to investigate the relationship between the number of activated EECT-lncRNAs and the methylation level of their promoters. Five cancer types were included in the analysis, with more than 100 platform-overlapped samples available. A significantly negative association between the average methylation level of all EECT-lncRNA promoters and the number of activated EECT-lncRNAs in each sample was observed, after adjustment for cancer type (Beta=-65.60, *P*=2.43×10^−18^) (Figure 5). The negative association remained (Beta=-83.03, *P*=2.16×10^−15^) after we included the methylation level of all gene promoters, suggesting that EECT-lncRNAs might be activated by promoter hypo-methylation.

## DISCUSSION

CT genes are a class of genes predominantly expressed in the germ cells of the testis and are ectopically reactivated in a wide range of tumor cells [2]. Accumulating evidence suggests that this group of genes can serve as epi-driver candidates, as their typical expression patterns show similarity with gametogenesis and tumorigenesis: immortalization of primordial germ cells and transformation of tumor cells; meiosis of spermatogonia and aneuploidy in tumor cells; migration of primordial germ cells and metastasis of tumor cells [2]. However, previous studies have focused on protein-coding genes. Our recent study proposed that non-coding RNAs exhibiting testis-specific expression patterns can also promote tumorigenesis [8]. Therefore, a systematic exploration of such lncRNAs could provide a critical step in unraveling the roles of CT-lncRNAs in driving the development of cancers. In the current study, we performed a comprehensive analysis of CT-lncRNAs and EECT-lncRNAs using multiple independent open-access databases. This study provides additional evidence for the driver roles of CT genes and for the idea that CT-lncRNAs/EECT-lncRNAs might also play important roles in the progression and maintenance of tumorigenesis.

Previous evolutionary analyses have shown that lncRNAs have undergone a low degree of constraint during the evolutionary process, as the transcripts of lncRNAs exhibited less evolutionary conservation than protein-coding genes [12] [13]. Interestingly, we found that both lncRNAs and protein-coding genes with testis-specific expression patterns seem to evolve more rapidly than those without (Supplementary Figure 2). Moreover, CT-lncRNAs are more prone to be located on chromosomes 17-19, which are reported under purifying selection [14-16]. Since the conservation of RNAs may depend more upon functional interactions with DNA, RNA or protein molecules rather than traditional sequence conservation [17, 18], we postulated that such weak evolutionary constraint of CT-lncRNAs/EECT-lncRNAs might be an indication of functionality in their secondary structure [18, 19].

Previously, we established a proteome profile for mouse spermatogonial stem cells (SSCs) and identified 682 proteins expressed in SSCs. Among their ortholog genes in humans (683), we observed a significant enrichment in CT genes (Supplementary Figure 6A). Interestingly, meiotically expressed genes [20] are also prone to exhibit a CT expression pattern (Supplementary Figure 6A), especially those in pachytene and post-meiotic phases (Supplementary Figure 6B). Since disorders of these phases can lead to incorrect non-crossover and crossover events [21, 22] and markedly affect genomic architecture [23], we proposed that the activation of CT genes may lead to genomic instability [24, 25]. While genes sharing similar functions or participating in the same biological pathway are assumed to exhibit similar expression patterns [26, 27], we speculated that activation of CT-lncRNAs may also destroy the maintenance of genome stability.

For a global view of the precise functions of CT-lncRNAs, we conducted a systematic lncRNA-coding gene co-expression analysis and assigned functions to all our defined CT-lncRNAs. Interestingly, we found a significant enrichment of CT-lncRNAs in cell cycle-related pathways (cell cycle, base excision repair, DNA replication). It is generally known that during the cell cycle process, various DNA damage, including both spontaneous and induced, and DNA repair processes occur [28] and that these will affect cell function and ultimately lead to aneuploidy and genome instability [29-31]. In our analysis, the activation of CT-lncRNAs was indeed found to be increasingly associated with the degree of genomic instability and the malignancy of tumors (Supplementary Figure 4, 5). While genome instability and its consequential aberrations can help tumor cells gain growth advantages and induce multiple hallmarks of cancer, CT-lncRNA activation may play a pivotal role in both the development and progression of cancers.

Similar to CT genes, testis-specific regulatory promoters and demethylation were also shown to be important mechanisms for the activation of CT-lncRNAs. More interestingly, we observed a significant enrichment of testis-specific enhancers upstream and downstream of TS-lncRNAs/CT-lncRNAs. Enhancers are regulatory elements positively activating the expression of target genes independent of orientation and distance [32], suggesting that adjacent enhancers might be another regulatory mechanism for TS-lncRNAs/CT-lncRNA expression. As several studies have proposed that a large proportion of lncRNAs are transcribed from enhancer elements [33] and are closely linked to the activity of nearby protein-coding genes, we attempted to clarify whether the adjacent enhancer elements also exerted effects on CT gene expression. As a result, TS-lncRNAs/CT-lncRNAs without enhancers nearby seem to be stronger regulators of their proximate testis-specific genes/CT genes (Supplementary Figure 5). Above all, despite testis-specific enhancers around CT-lncRNAs may be a novel mechanism for their activation; such elements do not seem to have an effect on nearby CT genes, in accordance with our previous result that no enrichment of enhancers was associated with CT genes.

In summary, we identified 1,325 CT-lncRNAs that exhibited extremely high expression patterns in 14 cancer types using multiple publicly available databases. LncRNAs with such expression patterns were found to correlate with the malignant potential of cancers and might be potential regulators of the reactivation of CT genes in tumorigenesis. This finding greatly broadens our understanding of CT-lncRNAs and provides new insight into the role of CT genes in driving carcinogenesis.

## MATERIALS AND METHODS

### Public databases used in this study

We utilized a recently constructed large-scale RNA-seq dataset, The Atlas of ncRNA in Cancer (TANRIC) [34], from the MD Anderson Cancer Center, to systematically explore CT-lncRNAs and extremely highly expressed CT-lncRNAs (EECT-lncRNAs) in tumor samples based on the testis-specific lncRNAs (TS-lncRNAs) defined in our previous study. TANRIC is a publicly available database based on the RNA-seq data of 20 cancer types retrieved from The Cancer Genome Atlas (TCGA) project. We obtained expression quantification data from http://ibl.mdanderson.org/tanric/_design/basic/index.html. Fourteen cancer types with more than 100 samples possessing expression data for both protein-coding genes and lncRNAs were included in this study. Ten of these cancer types, with more than 100 platform-overlapped samples, also possessed clinical information. The expression data of lncRNAs were quantified as RPKM. Detailed sample information for this database is listed in Supplementary Table 6. The information of other public databases used in this study, including the Encyclopedia of DNA Elements (ENCODE) project, the Functional Annotation of The Mammalian Genome (FANTOM) project and TCGA project, has been well described in our previous work [8].

### Definition of testis-specific lncRNAs

We previously analyzed the transcriptomics data of normal tissues (24 different organs from 175 individuals) from GTEx and used specificity measure to classify all human genes (50,016 genes) into six categories (C1-C6) based on their expression pattern, including high confidence testis-specific expressed protein coding genes (C1) and non-coding genes (C2), moderate confidence testis-specific expressed protein-coding genes (C3) and non-coding genes (C4), low confidence testis-specific expressed genes (C5), genes with transcripts exhibiting a testis-specific expression pattern (C6a) and genes without transcripts exhibiting a testis-specific expression pattern (C6b) [8].

According to GENCODE v19 annotation data, lncRNAs are reclassified into six biotypes [12], including 3 prime overlapping ncRNA (lncRNAs located within the 3′ UTR of protein-coding genes), antisense (lncRNAs overlapping any protein-coding genes on the opposite strand), lincRNA (long intergenic non-coding RNA with a length greater than 200 bp), sense intronic (lncRNAs located within the intron of any protein-coding gene), sense overlapping (lncRNAs within any protein-coding gene within its intron on the coding strand) and processed transcript (transcripts without an open reading frame). We first extracted 5,043 genes annotated as lncRNAs based on GENCODE v19 from our previously defined high- and median- confidence testis-specific expressed ncRNAs. LncRNAs with exons that overlapped with any known coding genes based on the gene annotations of GENCODE v19 and RefGene were further filtered. The remaining 3,302 lncRNAs were considered as TS-lncRNAs. Genes annotated as lncRNAs from the C6b group were defined as non-testis-specific lncRNAs (nonTS-lncRNAs).

### Criteria for the definition of CT-lncRNAs and EECT-lncRNAs

The above-defined TS-lncRNAs were further classified into CT-lncRNAs and EECT-lncRNAs. TS-lncRNAs meeting the following criteria were defined as CT-lncRNAs: (1) exhibited a testis-specific expression pattern with high confidence; and (2) exhibited expression (RPKM > 0.1) in at least 1% of the cancer samples. CT-lncRNAs that exhibited an extremely high expression pattern (EE: log_2_RPKM > Mean (log_2_RPKM)+3×SD(log_2_RPKM)) in at least 1% of the cancer samples were further defined as EECT-lncRNAs. When defining extremely high expression patterns, expression values of zero were set to one, and all the data were log2 transformed.

### In-house lung adenocarcinoma RNA-seq expression data

We obtained the lncRNA expression data of 24 lung adenocarcinoma patients from ArrayExpress (E-MTAB-4063). A detailed sample description was given in our previous study [8]. The data were quantified as normalized read counts (normalized by upper-quantile method) using RSEM (RSEM v1.2.12) [35]. To maintain consistency with our previous study, we defined CT-lncRNAs here as follows: (1) TS-lncRNAs that exhibited a testis-specific expression pattern with high confidence; and (2) TS-lncRNAs that exhibited expression (normalized read counts > 5) in at least 1% of the cancer samples. CT-lncRNAs exhibited an extremely high expression pattern (EE: log_2_ (normalized read counts) > Mean (log_2_ (normalized read counts))+3×SD(log_2_(normalized read counts))) in at least 1% of the cancer samples.

### Evolutionary conservation analysis

The evolutionary conservation was evaluated by the 46way vertebrate PhastCons conserved elements and the 46way vertebrate PhyloP scores. All the data were retrieved from the UCSC Genome Browser (hg19, http://genome.ucsc.edu/). We calculated the average PhastCons scores of the exons and promoter regions for both protein-coding genes and lncRNAs to represent the conservation level. Raw PhyloP scores represented the -log_10_ *P*-value of the likelihood ratio test under a null hypothesis of neutral evolution: a positive score indicated that the site was predicted to be more conserved than neutral, while a negative score indicated that the site was predicted to be less conserved. We re-computed the PhyloP scores using the following formulas to make the comparison easier. PhyloP_new_ =1-0.5×10^−PhyloPraw^if PhyloP_raw_ >0, and PhyloP_new_ =0.5×10^PhyloPraw^ if PhyloP_raw_ <0. The rescaled PhyloP scores ranged from 0 to 1, where a higher score indicated higher evolutionary conservation [36].

The exons were extracted from the GENCODE v19 transcripts annotation file, and the promoter regions were defined as regions 2 kb upstream and 0.5 kb downstream of the transcript start sites. The Wilcoxon rank sum test was performed to compare the average PhastCons scores and rescaled PhyloP scores between the CT-lncRNAs/EECT-lncRNAs and nonTS-lncRNAs, as well as CT genes/extremely highly expressed CT genes (EECTGs) and non-testis-specific gene (non-TSGs).

### Co-expression analysis

Since the expression of lncRNAs retrieved from the TANRIC database was quantified as RPKM and the original expression quantification from the TCGA projects was quantified as read counts, we could not evaluate the expression levels of coding genes and non-coding genes at the same level. Thus, we downloaded the raw Illumina HiSeq RNA sequencing files for the lung adenocarcinoma samples (n=487) and performed alignment and assembly. A standard STAR-HTSeq-DESeq2 pipeline was used to quantify gene expression, and both the reference genome annotation files and the transcriptome reference gene set were downloaded from the GENCODE v19 databases. The gene expression was quantified by normalized read counts. Co-expression analysis was performed with the Spearman rank-sum test to avoid the influence of skewness.

### Pathway enrichment analysis and functional annotation of lung adenocarcinoma specific CT-lncRNAs

To predict the function of lncRNAs based on their co-expressed protein-coding genes, only genes included in the Kyoto Encyclopedia of Genes and Genomes (KEGG) database [37] were included in this analysis. First, we filtered out genes that exhibited expression (normalized read counts >5) in less than 1% of the lung adenocarcinoma samples. Then, we conducted co-expression analysis of all lncRNAs and protein-coding genes included in the KEGG database and computed Spearman’s rank correlation coefficient for all lncRNA-protein-coding pairs; thus, a set of co-expressed protein-coding genes was obtained for each lncRNA (ranking the protein-coding genes by their correlation coefficient, and the leading 10% positively correlated and negatively correlated protein-coding genes were each selected) to perform pathway enrichment analysis. Enrichment analysis was conducted with the R Bioconductor package clusterProfiler, and the enrichment *P*-values were adjusted by the Benjamini-Hochberg false discovery rate (FDR-BH) for multiple test correction. Finally, each lncRNA was functionally annotated with significantly enriched KEGG pathways (*P*_BH_<0.01) among the co-expressed protein-coding genes. To avoid bias, we performed the same analysis for protein-coding genes not included in the KEGG database and expanded the annotated genes in this database. Given all the lung adenocarcinoma specific CT-lncRNAs, the pathway enrichment analysis was conducted for each KEGG term separately under a hypergeometric distribution (1) based on the updated annotation database.

Herein, *N* represents the number of all genes included in the KEGG database and the number of novel successfully annotated genes (lncRNAs and protein-coding genes); *M* represents the number of genes annotated in a specific KEGG term and the number of novel genes (lncRNAs and protein-coding genes) annotated in the same KEGG term; *n* represents the number of all successfully annotated CT-lncRNAs, and *m* represents the number of CT-lncRNAs annotated in a specific KEGG term.

### Evaluation of the correlation between mutations and EECT-lncRNAs

We utilized a significant mutated genes (SMGs) mutation ratio, which was defined in our previous work, to represent the degree of samples driven by SMGs. A linear regression model was used to evaluate the correlation between the SMG mutation ratio and the number of activated EECT-lncRNAs. In the TCGA lung adenocarcinoma data, 230 samples were divided into two parts. 143 samples harboring activating mutations in known lung cancer driver genes were defined as oncogene-positive samples, while 87 without such mutations were classified into the oncogene-negative group [38]. Wilcoxon’s rank-sum test was used to compare the number of activated EECT-lncRNAs between the two groups. Fisher’s exact test was used to evaluate the correlation between the mutation pattern of known lung cancer driver genes [38] and the expression pattern of EECT-lncRNAs.

### Evaluation of TS-lncRNAs/CT-lncRNAs related to testis-specific regulatory elements

We performed an enrichment analysis to evaluate the correlation between TS-lncRNAs/CT-lncRNAs and their nearby testis-specific regulatory elements with Fisher’s exact test. We included three types of testis-specific regulatory elements (promoters, methylation sites and enhancers) in this analysis. The definition and identification of testis-specific regulatory elements were described in detail in our previous study [8].

### Statistical analysis

Linear regression analysis was performed to evaluate the relationship between the number of activated EECT-lncRNAs or EECTGs in each tumor sample and AJCC tumor stage adjusted for age, gender and cancer types. The association between activated CT-lncRNAs/EECT-lncRNAs and CT genes/EECTGs was also analyzed with a linear regression model. All the enrichment analyses mentioned in this study were performed with Fisher’s exact test. General statistical analyses were performed with R (R version 3.2.2).

## SUPPLEMENTARY MATERIALS FIGURES AND TABLES















## Abbreviations

CT: (cancer-testis)
CT-lncRNA: (cancer-testis long non-coding RNA)
EECT-lncRNA: (extremely highly expressed CT-lncRNA)
KEGG: (the Kyoto Encyclopedia of Genes and Genomes)
lncRNA: (long non-coding RNA)
RPKM: (reads per kilobase per million mapped reads)
SMG: (significant mutated gene)
TANRIC: (The Atlas of ncRNA in Cancer)
TCGA: (The Cancer Genome Atlas)
TS-lncRNA: (testis-specific lncRNA)
