# Supplementary material for: Systematic identification of long non-coding RNAs with cancer-testis expression patterns in 14 cancer types
Source: Oncotarget. 2017 Oct 19;8(55):94769–79. doi: 10.18632/oncotarget.21930 (PMC5706911; doi:10.18632/oncotarget.21930)
Supplement: Supplementary file 1 [file oncotarget-08-94769-s001.pdf]

Systematic identification of long non-coding RNAs with cancer-testis expression patterns in 14 cancer types

SUPPLEMENTARY MATERIALS

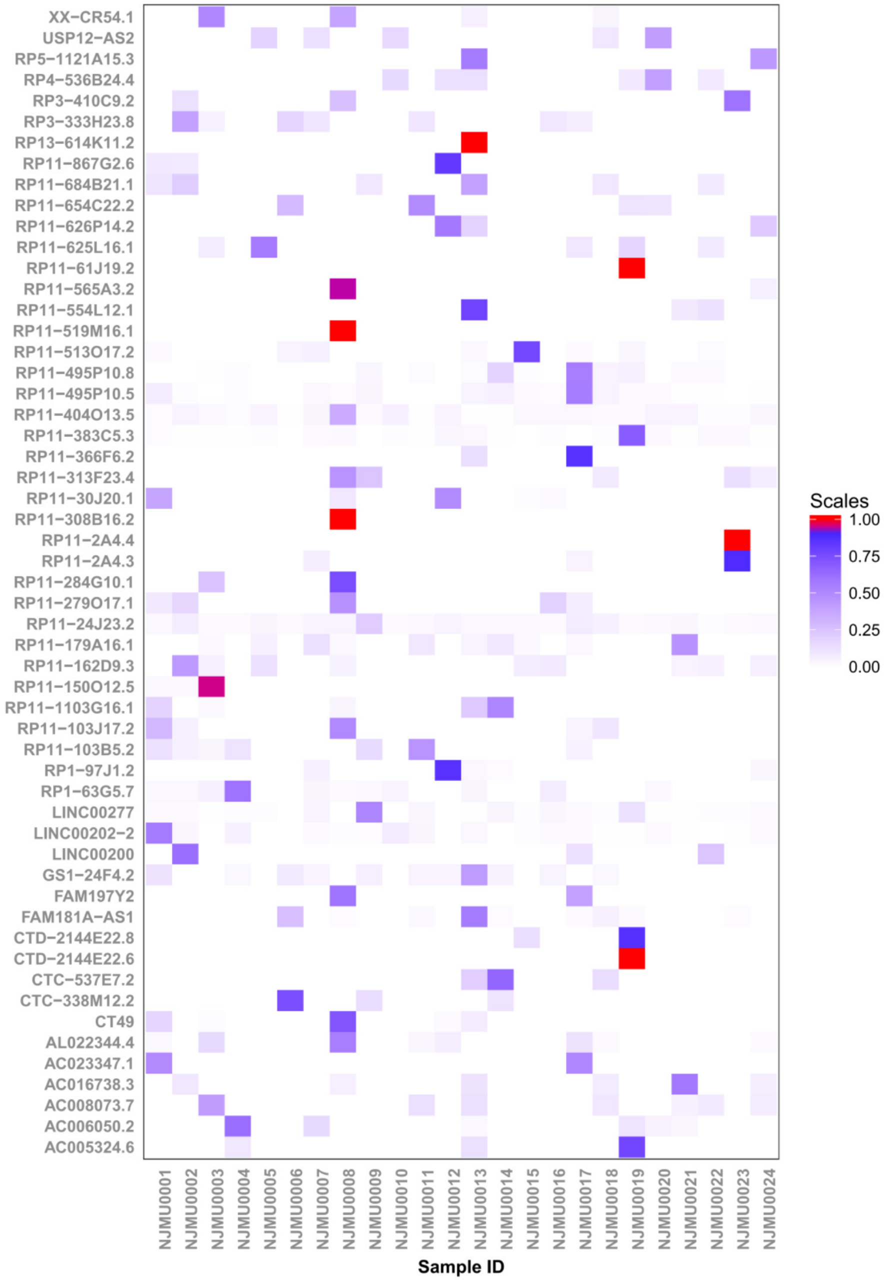

Supplementary Figure 1: Extremely high expression patterns of validated EECT-lncRNAs in our 24 lung adenocarcinoma samples. The red color represents extremely highly expressed samples, and blue represents other samples. The depth of color represents the expression level.

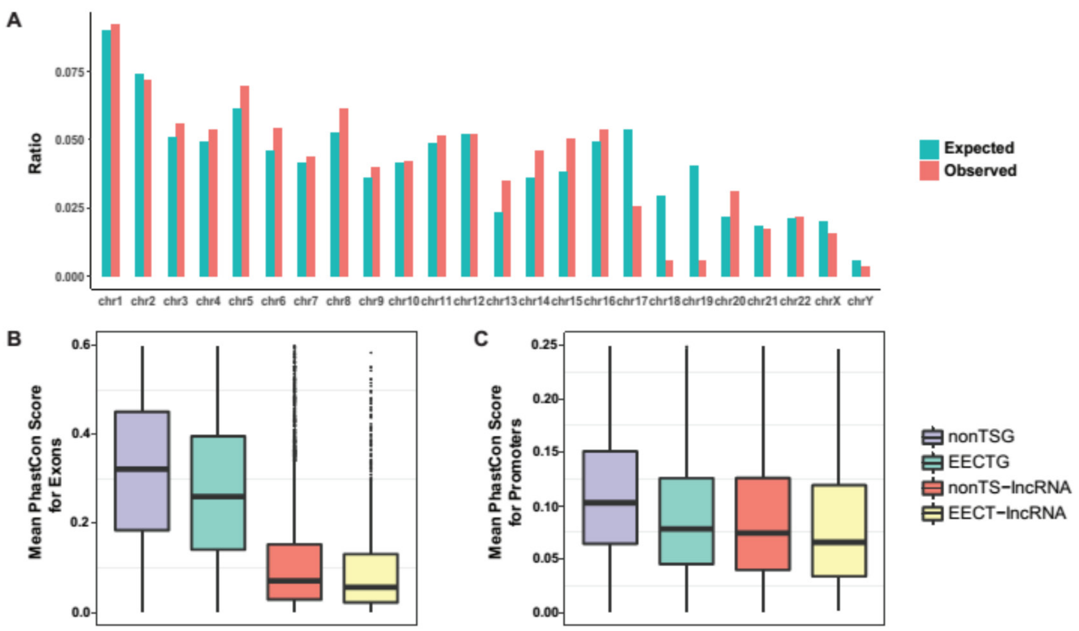

Supplementary Figure 2: General description of EECT-lncRNAs. **(A)** Chromosomal distribution of EECT-lncRNAs. **(B)** Distribution of PhastCons scores of exon sequence for nonTSGs, EECTGs, nonTS-lncRNAs and EECT-lncRNAs. **(C)** Distribution of PhastCons scores of promoter sequence for nonTSGs, EECTGs, nonTS-lncRNAs and EECT-lncRNAs.

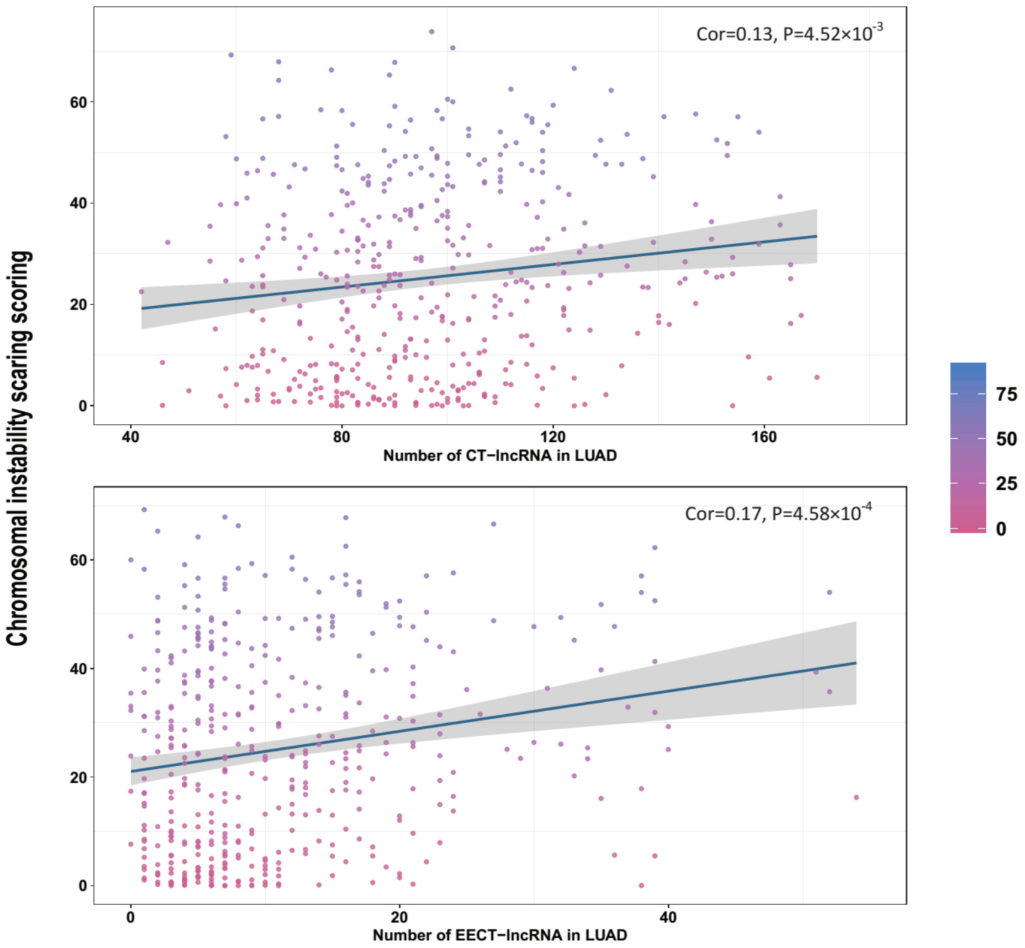

Supplementary Figure 3: Positive linear correlation between AICNA and the number of activated CT-lncRNAs/EECT-lncRNAs in TCGA lung adenocarcinoma samples.

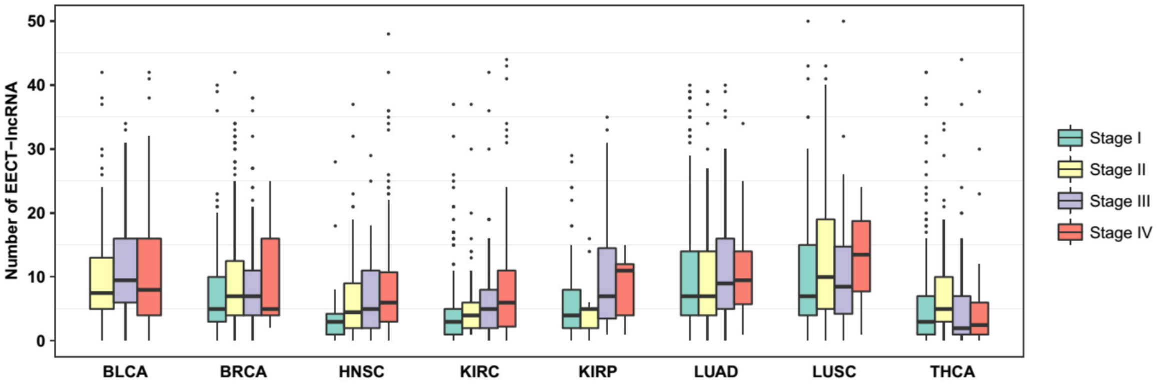

Supplementary Figure 4: Positive linear correlation between the number of activated EECT-lncRNAs and AJCC tumor stage.

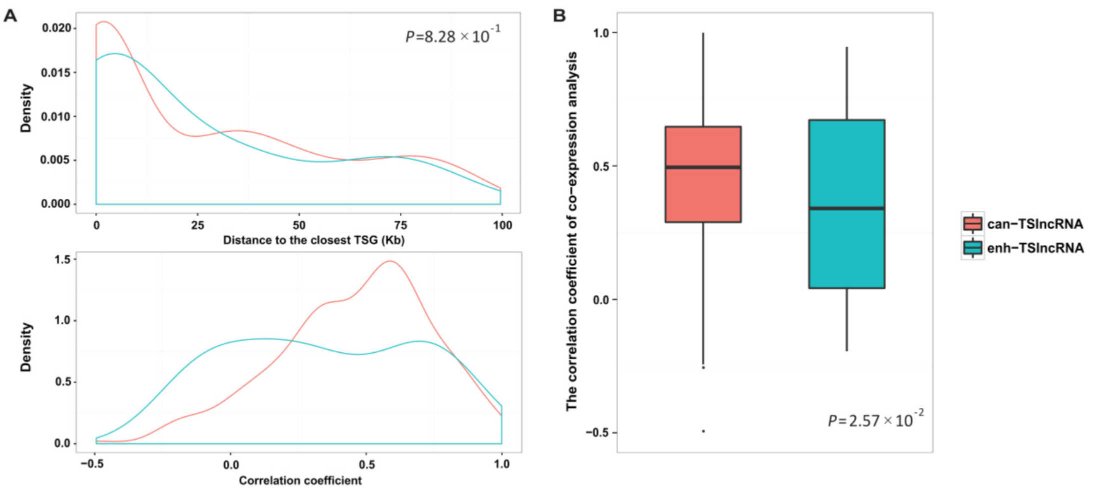

Supplementary Figure 5: Expression correlation coefficient between TS-lncRNAs and their proximate testis-specific genes. (A) Distribution of distance between the proximate testis-specific genes (TSGs) and TS-lncRNAs with enhancers nearby and those without. **(B)** The expression correlation coefficient of TS-lncRNAs with enhancers nearby was significantly lower than those without. The box plot displays the first and third quartiles (top and bottom of the boxes), the median (band inside the boxes), and the lowest and highest point within 1.5 times the interquartile range of the lower and higher quartile (whiskers).

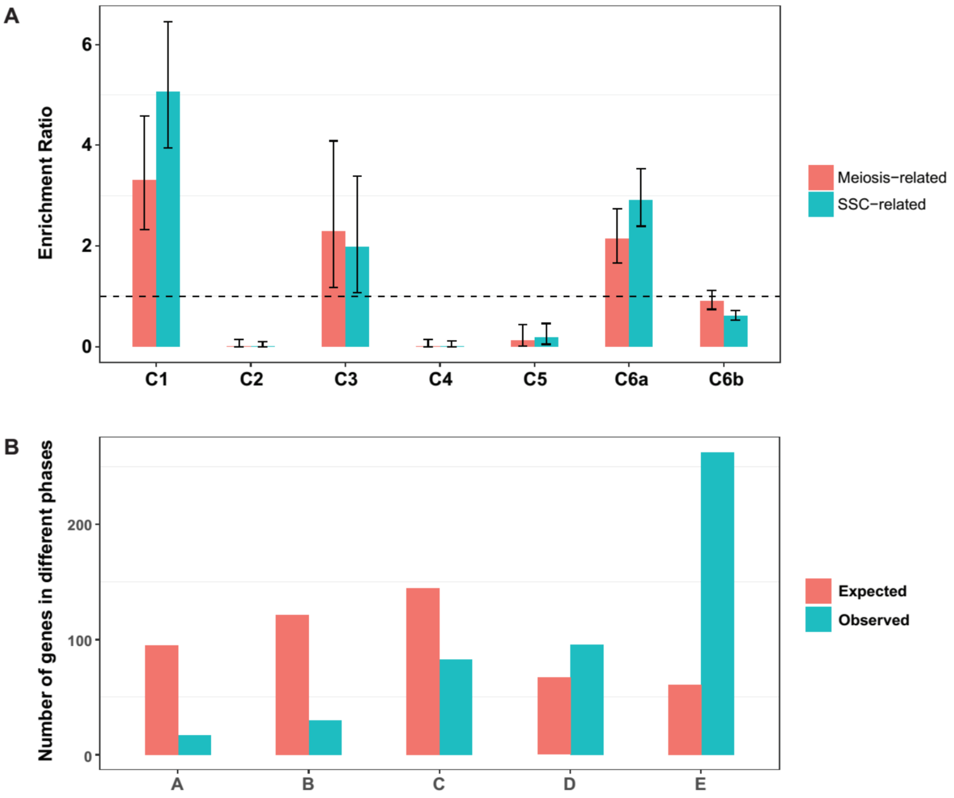

Supplementary Figure 6: Enrichment analysis of cell type-specific expressed genes. (A) Genes with CT expression pattern are significantly enriched in meiosis and SSS related genes. **(B)** CT genes are prone to participant in the pachytene and post-meiotic phases. A: promitive type A spermatogonia, Sertoli, Leydig, myoid B: types A and B spermatogonia, pre-leptotene and leptotene spermatocytes C: zygotene spermatocytes D: pachytene spermatocytes E: secondary spermatocytes, round and condensing spermatids

Supplementary Table 1: Information of CT-lncRNAs defined in our study: For each CT-lncRNA defined in our study, the percentage of expressed samples (RPKM>0. 1) and mean±SD of log2-transformed expression abundance are listed.

See Supplementary File 1

Supplementary Table 2: Information of EECT-lncRNAs defined in our study: For each CT-lncRNA defined in our study, the percentage of expressed samples (log2-transformed expression > (mean(log2-transformed expression)+3*sd(log2-transformed expression))).

See Supplementary File 2

Supplementary Table 3: Functional annotation of LUAD-specific CT-lncRNAs.

See Supplementary File 3

Supplementary Table 4: Pathway enrichement analysis results of LUAD-specific CT-lncRNAs.

See Supplementary File 4

Supplementary Table 5: EECT-lncRNAs are significantly different activated between oncogene-positive and oncogene-negative samples.

See Supplementary File 5

Supplementary Table 6: Information of the TCGA samples used in this study.

See Supplementary File 6
